# The effect of air gap on the total and local thermal insulation of Chinese male costumes from twenty minority ethnic groups

**DOI:** 10.1186/2046-7648-4-S1-A89

**Published:** 2015-09-14

**Authors:** Wen Shi, Hui Peng, Yehu Lu, Faming Wang

**Affiliations:** 1 Laboratory for Clothing Physiology and Ergonomics, the National Engineering Laboratory for Modern Silk, Soochow University, Suzhou, 215123, China

## Introduction

Clothing thermal insulation is an important thermo-physical parameter to determine thermal comfort. Previous studies have shown that clothing thermal insulation can be largely affected by the air speed, the body movement, the body posture, clothing physical properties and design features [1,2]. Air gaps trapped within the clothing microenvironment is one of the inherent factors that determine the thermal insulation of the clothing [3]. In this study, the effect of air gap on the total and local thermal insulation of 20 sets of Chinese male costumes was investigated by a 3D body scanner and thermal manikin measurements.

## Methods

A sample of twenty clothing ensembles of typical male ethnic costumes were selected from 20 Chinese minority groups. They were divided into 6 major groups based on their design characteristics. The total and local thermal insulation of those male costumes were measured on a 'Newton' thermal manikin, which was divided into 11 regions for data analysis. A constant surface temperature of 34.0 °C was used. All tests were conducted in a climatic chamber, where the air velocity was 0.4 (0.1) m.s^-1^. The test protocol strictly followed the ISO 15831 (2004) [4]. Three independent replications of tests for each ensemble were performed. The coefficient of variance of each test scenario should was within 10 %. To capture the 3D body shape and clothing air gap, a VITUS Smart 3D whole body laser scanner (Human Solutions GmbH, Kaiserslautern, Germany) was used. The nude manikin was first scanned, and then the dressed manikin was scanned with the same position. Each garment was scanned for three times to determine the reproducibility of the measurements. Nude and clothed scans were required to be overlapped and aligned as accurate as possible to calculate the thickness of clothing air gap by the Geomagic Qualify 12 software (Geomagic Inc., Morrisville, NC).

## Results

The total thermal insulation of the 20 sets of Chinese male ethnic costumes ranges from 0.81~1.48 clo (i.e., 0.125~0.230 m^2^·°C /W), and the average air gap thickness ranges from 12.67~51.39 mm. The local thermal insulation and air gap thickness of those ethnic costumes at different body regions differed distinctively (*p *= 0.05). Thermal insulation of the front torso is lower than that of the back; the local thermal insulation at the abdomen and the lower back regions are greater than those of the chest and the upper back regions. Local insulation was greatest at the pelvis region. Local air gap thickness was smallest at torso region and greatest at the legs. The local air gap thickness at the abdomen and the lower back regions are greater than those of the chest and the upper back regions.

## Discussion

Local insulation results are highly correlated with the uneven distribution of the clothing local air gap. Based on the data of the air gap distribution, the total and local thermal insulation of each ethnic costume, a scatter chart with markers was plotted for those 20 male ethnic costumes and a linear regression equation was developed accordingly. The total clothing thermal insulation prediction equation is *I_t_*= 0.0002*V_cl_*^2^+0.022*V_cl_*+0.503 (R^2 ^= 0.55), and the total clothing area factor prediction equation is *f_cl_*= 0.47*I_cl_+1.0 (R^2 ^= 0.54). The research findings contributed to the knowledge on clothing thermal comfort of Chinese male costumes, and hence provided a technical base for future thermal comfort studies.
